# Chromosome-level reference genome for North American bison (*Bison bison*) and variant database aids in identifying albino mutation

**DOI:** 10.1093/g3journal/jkad156

**Published:** 2023-07-22

**Authors:** Sam Stroupe, Carly Martone, Blake McCann, Rytis Juras, Helena Josefina Kjöllerström, Terje Raudsepp, Donald Beard, Brian W Davis, James N Derr

**Affiliations:** Department of Veterinary Pathobiology, Texas A&M University School of Veterinary Medicine and Biomedical Science, College Station, TX 77843, USA; Department of Veterinary Pathobiology, Texas A&M University School of Veterinary Medicine and Biomedical Science, College Station, TX 77843, USA; National Park Service, Theodore Roosevelt National Park, Medora, ND 58645, USA; Department of Veterinary Integrative Biosciences, Texas A&M University School of Veterinary Medicine and Biomedical Science, College Station, TX 77843, USA; Department of Veterinary Integrative Biosciences, Texas A&M University School of Veterinary Medicine and Biomedical Science, College Station, TX 77843, USA; Department of Veterinary Integrative Biosciences, Texas A&M University School of Veterinary Medicine and Biomedical Science, College Station, TX 77843, USA; Texas Parks and Wildlife, Caprock Canyons State Park & Trailway, Quitaque, TX 79255, USA; Department of Veterinary Integrative Biosciences, Texas A&M University School of Veterinary Medicine and Biomedical Science, College Station, TX 77843, USA; Department of Small Animal Clinical Sciences, Texas A&M University School of Veterinary Medicine and Biomedical Science, College Station, TX 77843, USA; Department of Veterinary Pathobiology, Texas A&M University School of Veterinary Medicine and Biomedical Science, College Station, TX 77843, USA

**Keywords:** North American bison, genomics, albinism, tyrosinase gene, bison reference genome, genome variant database

## Abstract

We developed a highly contiguous chromosome-level reference genome for North American bison to provide a platform to evaluate the conservation, ecological, evolutionary, and population genomics of this species. Generated from a F1 hybrid between a North American bison dam and a domestic cattle bull, completeness and contiguity exceed that of other published bison genome assemblies. To demonstrate the utility for genome-wide variant frequency estimation, we compiled a genomic variant database consisting of 3 true albino bison and 44 wild-type pelage color bison. Through the examination of genomic variants fixed in the albino cohort and absent in the controls, we identified a nonsynonymous single nucleotide polymorphism (SNP) mutation on chromosome 29 in exon 3 of the tyrosinase gene (c.1114C>T). A TaqMan SNP Genotyping Assay was developed to genotype this SNP in a total of 283 animals across 29 herds. This assay confirmed the absence of homozygous variants in all animals except 7 true albino bison included in this study. In addition, the only heterozygous animals identified were 2 wild-type pelage color dams of albino offspring. Therefore, we propose that this new high-quality bison genome assembly and incipient variant database provides a highly robust and informative resource for genomics investigations for this iconic North American species.

## Introduction

North American bison (*Bison bison*) are an iconic species due to their cultural and spiritual connection with Native American people, production potential to ranchers, and symbology of successful conservation efforts in federal and state parks and wildlife refuges. In addition, American bison were officially named the national mammal of the United States of America in 2016. Millions of bison once roamed the continent until they experienced a near extinction-level event reducing their census size by over 99% in the late 1800s (Hornaday 1889; Coder 1975). Their salvation is owed to various private herds established with wild caught calves across North America from Texas, USA to Alberta, Canada. Today, most bison are privately maintained as production livestock, while a smaller portion are managed as wildlife on public, nongovernmental organization, or tribal lands where they continue to play a vital role as grazers and food sources.

For more than 25 years, bison genetic research relied on mitochondrial genome sequences and microsatellite markers (Ward *et al*. 1999; Halbert and Derr 2007; Douglas *et al*. 2011; Forgacs *et al*. 2016). While these technologies were essential to laying a strong foundation for bison genetic research, they only portray a portion of the genomic story. Additionally, the domestic cattle (*Bos taurus*) reference genome has primarily been used for bison whole genome analyses (Stroupe *et al*. 2022). However, mapping sequencing data between species induces misalignment due to divergence or absence of species-specific haplotypes. In addition, correcting for gain and loss of sequence between species is hindered by differences in genome structure and incomplete modeling of orthology. There are 2 recently published bison reference genomes (Dobson *et al*. 2021; Oppenheimer *et al*. 2021) but each has significant shortcomings. For example, the Dobson *et al*. (2021) assembly has extremely low contiguity, and the Oppenheimer *et al*. (2021) assembly lacks the complete assembly of the X chromosome.

Advancements in genomic technologies and reductions in costs have allowed for consistent improvements in reference genomes. Contemporary genome assembly methods have been developed to construct highly contiguous reference genomes. One such method is the trio binning approach as described by Koren *et al*. (2018). In this method, parental species short-read sequences are used to separate long reads from a F1 hybrid into 2 haplotypes, which are then assembled independently. This approach was first used with an Angus (*Bos taurus taurus*) × Brahman (*Bos taurus indicus*) cross and has since been used to generate other highly contiguous reference genomes (Low *et al*. 2020; Rice *et al*. 2020; Bredemeyer *et al*. 2021).

Reference assemblies are essential for the advancement of genomic research. They allow for identifying intraspecific or interspecific variation which provides a more accurate understanding of evolutionary history and demography, as well as genotypes underlying phenotypic variation (Genereux *et al*. 2020). In concordance with this presented de novo genome assembly for bison, we provide a framework of how resources compiled using this genome can be used to identify variation associated with phenotypic traits and contribute to long-term conservation goals of this species.

Identifying variant(s) associated with a white pelage color phenotype, specifically albinism, in bison provides facile phenotypic identification, cultural significance, and aesthetic value. Pelage color variation and albinism genetics have been studied extensively with many genes associated with a white pelage color phenotype in a multitude of mammalian species. A characterized point mutation in premelanosome protein 17 (*PMEL17* or *SILV*) gene (c.64G>A) is associated with a dilution phenotype in Charolais cattle that causes a pale pelage color (Gutiérrez-Gil *et al*. 2007). This incomplete dominant mutation produces diluted or white pelage color when heterozygous or homozygous, respectively. This phenotypic effect has also been observed in bison-Charolais hybrid offspring. Mutations in the *KITLG* gene confer white pelage color in horses and white spotting in mammals including horses (Haase *et al*. 2007), cattle (Brenig *et al*. 2013; Jivanji *et al*. 2019), and yaks (Zhang *et al*. 2014). Mutations in tyrosinase (*TYR*) have been shown to cause albinism in many mammalian species, including cattle (Schmutz *et al*. 2004), water buffalo (Damé *et al*. 2012), cats (Imes *et al*. 2006), humans (Stenson *et al*. 2003; Kamaraj and Purohit 2014), and mice (Hubbard *et al*. 2010). Leucistic animals that lack pheomelanin also have white pelage, in the case of white tigers, it is due to a mutation in the *SLC45A2* gene (Xu *et al*. 2013). Other genes such as *MITF* (Jivanji *et al*. 2019), *MLPH* (Li *et al*. 2016), and *MC1R* (Almathen *et al*. 2018) are also associated with a white or diluted pelage color.

**Fig. 1.**
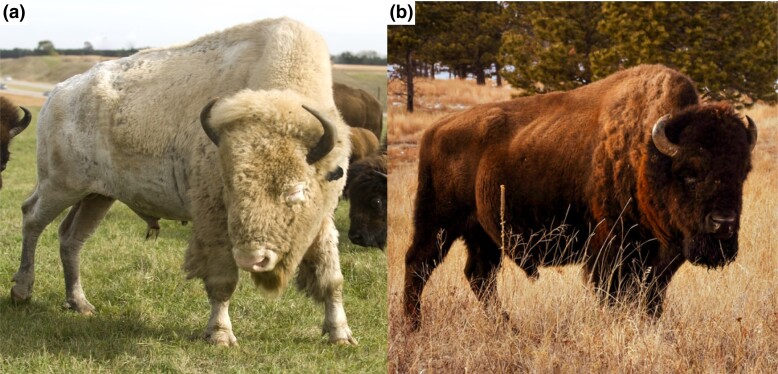
Pelage color variation in *Bison bison.* a) Albino bison. Note the white pelage, hypopigmented eyes, and hypopigmented skin particularly around eyes and nose. Photo courtesy of the National Buffalo Museum and Searle Swedlund of Jamestown, ND. b) Typical wild-type bison pelage color.

While white pelage is associated with various mutations across several genes in mammalian species, albinism has additional associated phenotypes. It is characterized by a decrease or absence of both pheomelanin and eumelanin in hair, skin, and/or eyes (Fig. 1) (Kinnear *et al*. 1985). In ocular or oculocutaneous albinism, nystagmus, photophobia, and reduced vision or blindness are frequently seen. Hypopigmentation of the skin also leaves the animal more susceptible to solar erythema (sunburn) as well as basal and squamous cell carcinomas (Oetting and King 1999). The most severe form of the phenotype, exhibiting complete or near complete hypopigmentation, is oculocutaneous albinism type 1 (OCA1) (King *et al*. 2003; Grønskov *et al*. 2007; Dolinska *et al*. 2017). OCA1 is commonly caused by a mutation in the tyrosinase (*TYR*) gene. Physiologically, the protein tyrosinase converts tyrosine to L-dihydroxyphenylalanine (DOPA) which is converted to dopaquinone, the latter of which is converted to melanin (Kinnear *et al*. 1985). Thus, the loss-of-function of tyrosinase activity results in lack of melanin as seen in OCA1. White bison samples used in this study were suspected to be afflicted with albinism, due to physical characteristics of white hair and lack of epidermal and ocular pigment (Fig. 1).

Here, we present a highly contiguous single-haplotype reference genome for the North American bison. This reference genome was used to create a variant catalog for the identification of a point mutation in the *TYR* gene that is associated with albinism and is identical to a causative mutation for OCA1 in the human ortholog.

## Materials and methods

### Biological material

Samples used for the genome assembly consist of a female bison (*B. bison*) “Molly” (SAMN33913325), a male domestic cattle angus-cross (*B. taurus*) herd representative (SAMN33913326), and a female F1 bison-cattle offspring “Midnight” from a private ranch in Texas (SAMN33913332). Biopsy tissue samples were collected from the bison dam and domestic cattle bull. Fresh blood and sterile ear punch samples were collected from the F1 bison-cattle hybrid. A cell line was established from the F1 hybrid and karyotyped by the Molecular Cytogenetics Laboratory at Texas A&M University School of Veterinary Medicine to confirm species identification (Supplementary Fig. 1). Additionally, a sterile ear punch sample was collected from a separate privately owned female bison to establish a primary fibroblast cell line for generating Bionano optical mapping data.

Hair, blood, and/or tissue samples of bison used for this study were obtained during the annual roundups by the ranchers responsible for the management or ownership of the herds. One tissue sample was collected postmortem by the herd manager. Additionally, bone and hide samples were donated from their curated collection. All samples were collected by or under the direction of the owner or ranch manager in charge of each bison herd.

### Cell cultures and chromosome preparations

Metaphase chromosome spreads were prepared from primary fibroblast cultures using standard procedures (Raudsepp and Chowdhary 2008). Briefly, primary fibroblasts were grown in alpha MEM with nucleosides and Glutamax (Gibco), supplemented with 20% fetal bovine serum (FBS) at 5% CO_2_ to semi-confluency (∼60–70%). Metaphase cells were harvested with demecolcine solution (10 µg/ml; Sigma-Aldrich), treated with Optimal Hypotonic Solution (Rainbow Scientific), and fixed in 3:1 methanol/acetic acid. The cells were dropped on clean, wet glass slides and checked under phase contrast microscope (×200) for quality. Chromosomes were stained by GTG-banding (Seabright 1971) for karyotyping. Karyotyping and chromosome analysis was done with an Axioplan2 microscope (Carl Zeiss, Inc., Jena, Germany) and IKAROS (MetaSystems GmbH, Altlussheim, Germany) software. The chromosomes were identified and arranged into karyotypes according to the International System of Cytogenetic Nomenclature of the Domestic Bovids (Cribiu *et al*. 2001).

### Genome assembly

#### Long-read sequencing (Pacific Biosciences)

High molecular weight DNA was isolated from leukocytes, of the F1 bison-cattle hybrid (SAMN33913332), using the Nanobind CBB Big DNA Kit (Circulomics, Baltimore, MD) to yield high molecule length DNA. Four single molecule real-time (SMRT) cells were run in Circular Consensus Sequence (CCS or Hifi) mode and generated a total average sequence depth of 120×, given an approximately 3 Gb genome.

#### Short-read sequencing (Illumina)

DNA was extracted from the tissue, hair, and blood samples using the DNeasy or Gentra Puregene kit (Qiagen) according to manufacturer protocol (Supplementary Table 2). A total of 150 bp, paired-end libraries were subsequently prepped using the 2S Turbo library kit (Swift Biosciences). For bone and hide samples, DNA was extracted in a dedicated ancient DNA laboratory using the appropriate sterile techniques and equipment. Extraction was carried out following the Dabney extraction protocol (Dabney *et al*. 2013) but with the addition of a 30-min predigest stage (Damgaard *et al*. 2015). Libraries were subsequently prepped using the Accel-NGS 1S Plus DNA library kit (Swift Biosciences). All new short-read, whole genome data was sequenced using NovaSeq 6000 Illumina Next-Generation Sequencing by Texas A&M Institute for Genome Sciences and Society (College Station, TX).

### De novo assembly

The F1 hybrid Hifi sequence reads were sorted into haplotype contigs with Canu v2.1.1 using the trio binning assembly approach (Koren *et al*. 2018) using a female bison (*B. bison*) “Molly”, Angus-cross domestic cattle bull (*B. taurus*), and female F1 hybrid “Midnight” (Fig. 4). The bison (maternal) haplotype was then assembled to chromosome level using the scaffolding and chromosome identification methods as described below.

### Scaffolding and chromosome assignment

Chromatin conformation capture (Hi-C) data was previously generated by DNA-Zoo and downloaded from NCBI, accession SRR8616930 (Rao *et al*. 2014; Dudchenko *et al*. 2017). The raw Hi-C fastq reads were aligned to the bison haplotype contigs using BWA-mem (Li and Durbin 2009). Contigs were scaffolded with SALSA v2 using the default recommendations allowing the aligned Hi-C data to correct input assembly errors (Ghurye *et al*. 2019).

Next, Bionano optical mapping was used to further improve scaffolds. Optical mapping data was generated and analyzed according to the established Saphyr workflow (Bionano Genomics). Briefly, high molecular weight DNA was extracted from a female bison fibroblast cell line according to Bionano Genomics: Bionano Prep Cell Culture DNA Isolation Protocol. Once the DNA concentration was even throughout the solution and within the target parameters, it was labeled with Direct Label Enzyme (DLE-1) as stated in the Bionano Prep Direct Label and Stain (DLS) Protocol. The generated Bionano data was used assemble the haplotype with the options set to keep conflicts for both the Bionano and sequence assemblies and trimming overlapping sequence contigs turned off.

The BioNano Super-Scaffolds were aligned to *Bos taurus* ARS-UCD1.2 using MUMmer4's nucmer4 (Marçais *et al*. 2018). The genome-to-genome alignment was then visualized with Dot (https://dot.sandbox.bio). The ARS-UCD1.2 (*Bos taurus*) chromosomes were then aligned to each corresponding Bison scaffolds using D-Genies, minimap2 option, to orient and order the bison scaffolds into chromosomes (Cabanettes and Klopp 2018).

**Fig. 2.**
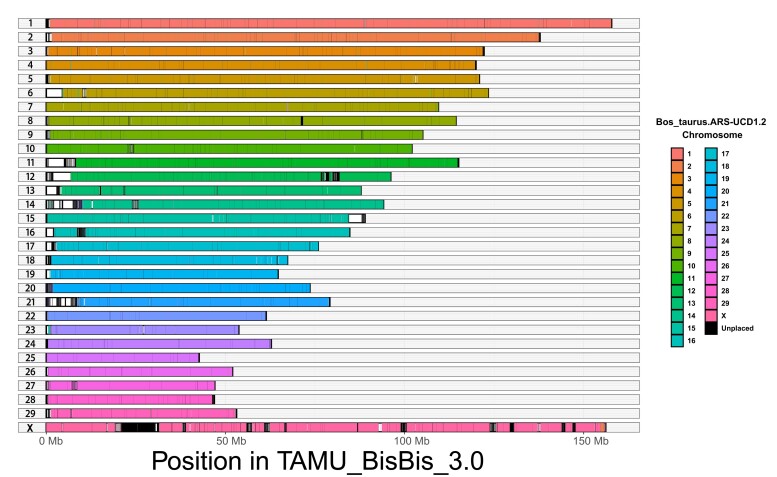
Genome alignment between TAMU_BisBis_3.0 and Bos_taurus_ARS-UCD1.2. Positions of conserved synteny between bison and cattle are highlighted based on chromosome. Regions aligned to unplaced scaffolds are indicated in black and sections without conserved synteny are indicated in white. Alignment statistics and structural variation identified are in Supplementary Table 1.

RagTag v2.0.1 was then used to anchor chromosome-level scaffolds using *Bos taurus* ARS-UCD1.2 as a reference (Alonge *et al*. 2021). Finally, the bison haplotype assembly was manually curated to correctly format, orient, and name each scaffold. The final bison haplotype assembly TAMU_BisBis_3.0 and *Bos taurus* ARS-UCD1.2 were aligned with minimap2 v2.17 to evaluate assembly completeness and cross-species genome comparison (Li 2021). The R package pafR v0.0.2 was used to visualize the genome synteny and variation with a coverage plot (Fig. 2) (Winter 2020).

### Annotation

A liftover annotation was performed using Liftoff v1.6.3 to annotate the bison genome using the *Bos taurus* ARS-UCD1.2 genome assembly gene model (Shumate and Salzberg 2021). Default parameters were used with the option to find additional gene copies with a sequence identity threshold of over 95% (script arguments; -copies –sc 0.95).

### Albinism variant identification

We used 47 bison whole genome sequences, including 3 from albino bison, to identify alleles associated with albinism. Bison whole genome sequence data was obtained as described above, as described by Stroupe *et al*. (2022), or downloaded from the National Center for Biotechnology Information (NCBI) (Supplementary Table 2). Sequence data was aligned to our bison genome using Burrows–Wheeler Aligner, bwa-mem2 v2.2.1, (Li and Durbin 2009; Vasimuddin *et al*. 2019) and sorted using SAMtools v1.12 (Li *et al*. 2009). Variants were called using GATK v4.1.0.0 HaplotypeCaller per chromosome, merged using CatVariants and CombineGVCFs options, then genotyped using GenotypeGVCFs according to GATK best practices (van der Auwera *et al*. 2013). Per individual number of sites, mean depth, and levels of heterozygosity were calculated using VCFtools v0.1.16, as were the delta allele frequency for all single nucleotide polymorphisms (SNPs) (Danecek *et al*. 2011).

SNP variant frequency was compared between 3 albino individuals and a cohort of 44 bison with wild-type pelage coloration. Under the hypothesis that albinism would be inherited as a monogenic recessive requiring a homozygous variant to exhibit albinism as is seen in other mammals, and that heterozygous variation may result in partial melanism, we calculated the delta allele frequency between albino and wild-type brown for each SNP. This identified 3,936 variants fixed for the alternate allele in albino but were fixed for the reference in all brown individuals, and 4,831 fixed for the alternate allele in brown but were fixed for the reference in albino. Fixed variants identified using allele frequency were filtered based on genomic context. VCFtools v0.1.16 was used to filter the albino alternate allele fixed sites using a missingness cutoff of 0.5 and a mean site depth of 5 leaving 180 variant sites due to low sequencing depth of some genomes. SNPeff 5.1d was used to evaluate these variants for potential amino acid alterations based on the liftover annotation (Cingolani *et al*. 2012).

AlphaFold v2.1.2 was used for predictive modeling of the protein given the amino acid replacement in position 372 (Jumper *et al*. 2021; Varadi *et al*. 2022). A fasta file of the amino acid sequence with the identified substation was used an input for AlphaFold using the suggested parameters. AlphaPickle v1.4.1 was used to create a graph for each model (.pkl) file (Arnold 2021). The predictive model and reference model from the database were visualized with the Protein Data Bank 3D Viewer (rcsb.org/3d-view).

### Albinism variant population frequency validation

A TaqMan SNP Genotyping Assay was designed for the identified SNP in the *TYR* coding region through ThermoFisher Scientific's Custom TaqMan Assay Design Tool. Four bison with whole genome sequencing data were genotyped with the developed TaqMan assay confirming cross platform validation of this SNP. In subsequent assays, a known albino and known homozygous wild-type sample were used as controls. A total of 283 bison, 7 known albino bison, and 276 wild-type bison were SNP genotyped to validate and help establish allele frequencies (Supplementary Table 3). The 2 individuals that were heterozygous at this SNP were dams of 2 of the albino bison included in this study. The owners of each set of mother–offspring identified the relationship between the individuals. Both dams of albino offspring qualified as parents with a panel of microsatellites used for parentage determination as described by Schnabel *et al*. (2000).

## Results and discussion

The presented de novo reference genome for the bison haplotype is a highly contiguous and complete assembly compared to other available genomes for bison, rivaling those of human, mouse, cattle, dog, and domestic cat (Table 1). In comparison to the current cattle reference genome (ARS_UCD_1.2), the presented bison genome shows very highly conserved synteny (Fig. 2), though considerable structural variation exists between species (Supplementary Table 1). The comparison between the 2 genomes revealed regions in this bison genome that are not represented in the cattle genome. However, the biological importance of these species level differences is not known, and they may be an artifact of unassembled sequence that was resolved with our assembly approach. Additionally, our assembly was able to place genomic regions that are unplaced in the ARS_UCD_1.2 cattle assembly. This can be particularly seen on the X chromosome where our approach was able to span gaps where only unplaced scaffolds of the cattle reference aligned (Fig. 2).

**Table 1.** Comparative genome assembly statistics.

Assembly	Species	Scaffold count	N50 (Mbp)	Haploid chromosome number	L50	Total length (bp)
TAMU_BisBis_3.0	*Bison bison* (American bison)	662	96.13	30	13	2,983,386,259
ARS-UCSU_bison1.0	*Bison bison* (American bison)	774	87.75	30	12	2,651,593,212
Bison_UMD1.0	*Bison bison* (American bison)	128,431	7.19	30	116	2,828,031,685
ARS-UCD1.2	*Bos taurus* (domestic cattle)	2,211	103.31	30	12	2,715,853,792
GRCh38.p14	*Homo sapiens* (human)	473	67.79	23	16	3,099,734,149
T2T-CHM13	*Homo sapiens* (human)	24	154.26	23	9	3,117,275,501
GRCm39	*Mus musculus* (house mouse)	102	106.15	20	11	2,728,222,451
F.catus_Fca126_mat1.0	*Felis catus* (domestic cat)	71	148.49	19	7	2,425,747,038
ROS_Cfam_1.0	*Canis lupus familiaris* (dog)	376	64.04	39	15	2,396,858,295

Genome assembly statistics for this new bison assembly compared to previous bison and other mammalian genome assemblies.

The 47-sample variant database consisted of 57,238,711 sites including 45,651,300 SNPs and 11,587,411 indels less than 500 bp pre-filtering. A total number of sites, mean depth, and measures of heterozygosity were calculated on a per individual basis (Supplementary Table 2). The 3 albino whole genome samples had a higher inbreeding coefficient, F, of 0.26, 0.33, and 0.40 compared to other modern bison samples of comparable coverages which averaged 0.16 (Supplementary Table 2).

**Fig. 3.**
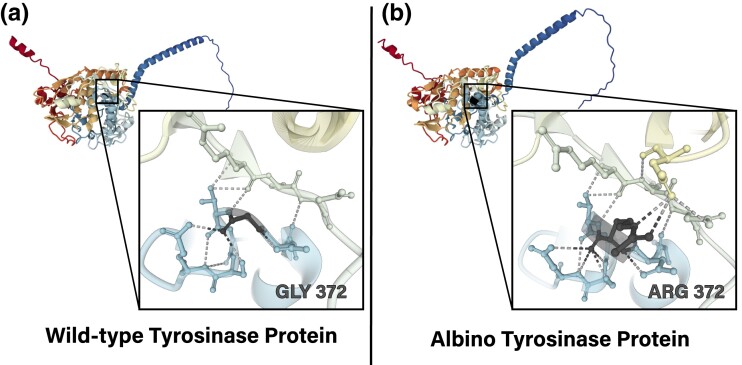
AlphaFold 3D models of tyrosinase protein comparing wild-type and albino forms. These models compare the reference wild-type a) to the albino b) protein conformations. The dark gray colored amino acid, at position 372, is the change due to the nonsynonymous mutation occurring in the albino bison. The boxed section shows local predicted interactions with the amino acid at position 372 (Arnold 2021; Jumper *et al*. 2021; Varadi *et al*. 2022).

There were 3,936 variants that were fixed for the alternate allele in albino bison and absent in wild-type bison. After filtering for depth and missingness, among the albino samples, 180 variants remained. Of these, only 1 nonsynonymous SNP was identified, a missense mutation at the base position 7,995,584 on chromosome 29, located in exon 3 of the *TYR* gene (c.1114C>T). The transition from cytosine to thymine in the first position of the codon causes an amino acid substitution of glycine for arginine at the protein position 372 (Fig. 3). Per our allele frequency calculations, the thymine allele was confirmed to be homozygous in all albino bison while absent in wild-type bison whole genome sequences. As seen in Fig. 3, the replacement of glycine with arginine more than doubles the molecular weight of the amino acid in position 372 altering the predicted amino acid interactions within that region. Therefore, the p.Gly372Arg substitution was suggested to cause a disruption in tyrosinase protein inducing a lack of melanin production and albino phenotype in some white bison.

**Fig. 4.**
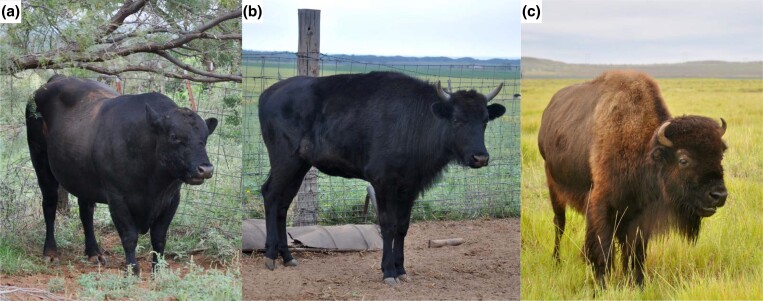
Parents and offspring for the trio binning genome assembly. a) Domestic Angus-cross bull, b) female F1 hybrid “Midnight”, and c) bison cow “Molly” used for genome assembly of North American bison.

A TaqMan SNP Genotyping Assay was designed and used to test 4 additional albino bison for this mutation and confirm genotypes for the 3 albino bison included in the whole genome sequencing. All 7 of the albino bison were homozygous for the putative causative mutation. Additionally, 276 wild-type bison from 29 bison populations, including 16 public and 13 private herds, were also genotyped at this SNP (Supplementary Table 3). Of the wild-type bison, 2 were heterozygous. These individuals were identified as the dams of albino calves by the owner and qualified as parents using a microsatellite parentage panel (Schnabel *et al*. 2000). All other wild-type bison tested were homozygous for the reference cytosine allele. A total of 326 bison were genotyped at this site, including 43 whole genome sequences, 279 TaqMan assay, and 4 genotyped on both platforms.

Recently, 2 bison reference genomes were published, however, both genome builds have their own limitations (Dobson *et al*. 2021; Oppenheimer *et al*. 2021). The first bison reference genome assembly, Bison_UMD1.0, was completed using short-read sequencing resulting in a highly fragmented genome assembly (Dobson *et al*. 2021). The second bison genome, ARS-UCSC_bison1.0, was assembled utilizing trio binning with a F1 hybrid long-read sequences and parental short-read sequencing, similar to this study (Oppenheimer *et al*. 2021). However, the long-read sequencing method, Oxford Nanopore Technologies, that was used for the ARS_UCSC_bison1.0 assembly is more error prone than the technology used for our methods, Pacific Biosciences (Weirather *et al*. 2017). Moreover, the F1 hybrid and bison parent were males, so the ARS-UCSC_bison1.0 haplotype was constructed without an X chromosome. The new bison genome assembly presented here is an improvement over previous assemblies and is the first complete chromosome-level assembly for North American bison.

Prior to this study, *TYR* was identified as an albinism-related gene in cattle (Schmutz *et al*. 2004), water buffalo (Damé *et al*. 2012), cats (Imes *et al*. 2006), humans (Kamaraj and Purohit 2014), mice (Hubbard *et al*. 2010), and other mammals. In cattle, a cytosine insertion resulting in a premature stop codon (nonsense mutation) at residue 316 of tyrosinase was reported to be one cause of albinism (Schmutz *et al*. 2004) though other genes have been identified conferring different forms of albinism including *MITF* (Wiedemar and Drögemüller 2014) and *SLC45A2* (Rothammer *et al*. 2017). In humans, 7 different types of albinism are currently recognized with causative mutations in *TYR* categorized into OCA1 (Aamir *et al*. 2021). According to the Human Gene Mutation Database (HGMD), there are over 400 characterized mutations in *TYR*, many of which are associated with albinism (Stenson *et al*. 2003). Of particular interest to this study is an OCA1A *TYR* mutation identified in a Brahmin family in Eastern India (Chaki *et al*. 2006). The SNP occurs at the same base position in the human ortholog as our identified bison mutation (c.1114), resulting in the same p.Gly372Arg substitution. Interestingly, this amino acid change is located within the copper-binding B (CuB) region of the tyrosinase protein where one of the most important sites of glycosylation is located (Olivares *et al*. 2003). Without this glycosylation event, tyrosinase cannot move out of the endoplasmic reticulum and therefore fails to catalyze the conversion of tyrosine to L-dihydroxyphenylalanine (DOPA) as previously described (Kinnear *et al*. 1985). Thus, this loss-of-function mutation in the tyrosinase gene truncates the biochemical pathway to melanin producing the albinism phenotype.

The observation of a mutation identified in exon 3 of the *TYR* gene on chromosome 29 (c.1114C>T) in 7 albino bison, shown in the heterozygous state in the dams, and absent in 317 bison with a wild-type pelage color is suggestive of this SNP being the causative mutation in these animals. These results agree with an autosomal recessive inheritance of oculocutaneous albinism type 1 with the known dams shown as carriers for albinism, similar to what is seen in humans (King *et al*. 2003; Dolinska *et al*. 2017). Further evidence supporting this mutation being causative for albinism in these bison comes from the fact that the same loss-of-function mutation is known to cause albinism in humans (Chaki *et al*. 2006). With a limited number of albino bison tested, it is possible that other mutations exist that explain cases of albinism observed in other white bison.

With the discovery of an albinism-associated mutation, we have provided a framework to use this genomic resource to uncover the genetic bases for important phenotypes in bison. Bison are a valuable species as both wildlife and livestock and therefore, many groups benefit by further understanding bison genomics. One such relative issue is immune response. Since bison and domestic cattle have been in proximity since domestic cattle's arrival to North America, many diseases are transmitted between the 2 species. However, in some cases, these species are known to react different to both pathogens and vaccines (Tessaro 1989; Berezowski *et al*. 2019). This has led to some highly debated issues such as those dealing with brucellosis caused by *Brucella abortus* in Yellowstone National Park (Meagher and Meyer 1994; Rhyan *et al*. 2001). Development of this bison specific genomic resource will assist future research focused on identifying genetic factors that contribute to traits of interest.

This high-quality annotated bison genome will also facilitate multiple arenas of future bison genetic research. For example, bison specific whole genome approaches will be possible without the potential biases inherent when using the cattle reference genome. In addition, population genetics studies will no longer need to rely on limited microsatellites (Schnabel *et al*. 2000; Halbert and Derr 2007) or solely mitochondrial genomes (Ward *et al*. 1999; Douglas *et al*. 2011; Forgacs *et al*. 2016). This high-quality bison reference genome also provides expanded opportunities for investigating genomic selection following the well-documented domestic cattle introgression experiments of the late 1800s (Stroupe *et al*. 2022). Finally, the availability of this completed chromosome-level genome will provide a platform to further define the evolutionary history and clade placement of species among the tribe Bovini. Overall, we expect that this genome assembly will greatly assist the conservation, ecological, evolutionary, and population genomics investigations of this iconic North American species.

## Supplementary Material

jkad156_Supplementary_Data

## Data Availability

The genome assembly and raw sequencing data are available at NCBI under the BioProject accession PRJNA945428, and gene annotation for TAMU_BisBis3 in GTF format and biallelic population SNV in VCF format can be found at Davis (2023). The short-read sequencing data accession numbers are listed in Supplementary Table 2. Albino samples are indicated in Supplementary Table 2 in the population column. TaqMan assay genotype data per population can be found in Supplementary Table 3. All additional necessary information can be found within the article, figures, tables, and at G3 online. Supplemental material available at G3 online.
