# Supplementary material for: Chromosome-level reference genome for North American bison (Bison bison) and variant database aids in identifying albino mutation
Source: G3 (Bethesda). 2023 Jul 22;13(10):jkad156. doi: 10.1093/g3journal/jkad156 (PMC10542314; doi:10.1093/g3journal/jkad156)
Supplement: jkad156_Supplementary_Data [file jkad156_supplementary_data.zip › Figure_S1_Legend_G3-2023-404214.docx]

**Figure S1: Karyotype of female bison x domestic cattle F1 hybrid, “Midnight”.**

GTG-banded karyotype and corresponding metaphase spread (below) of cattle x bison F1 hybrid. Chromosomes are arranged into the karyogram following the International System for Chromosome Nomenclature of Domestic Bovids (Cribiu et al. 2001).
