# Supplementary figures and images for: Chromosome-level reference genome for North American bison (Bison bison) and variant database aids in identifying albino mutation
Source: G3 (Bethesda). 2023 Jul 22;13(10):jkad156. doi: 10.1093/g3journal/jkad156 (PMC10542314; doi:10.1093/g3journal/jkad156)

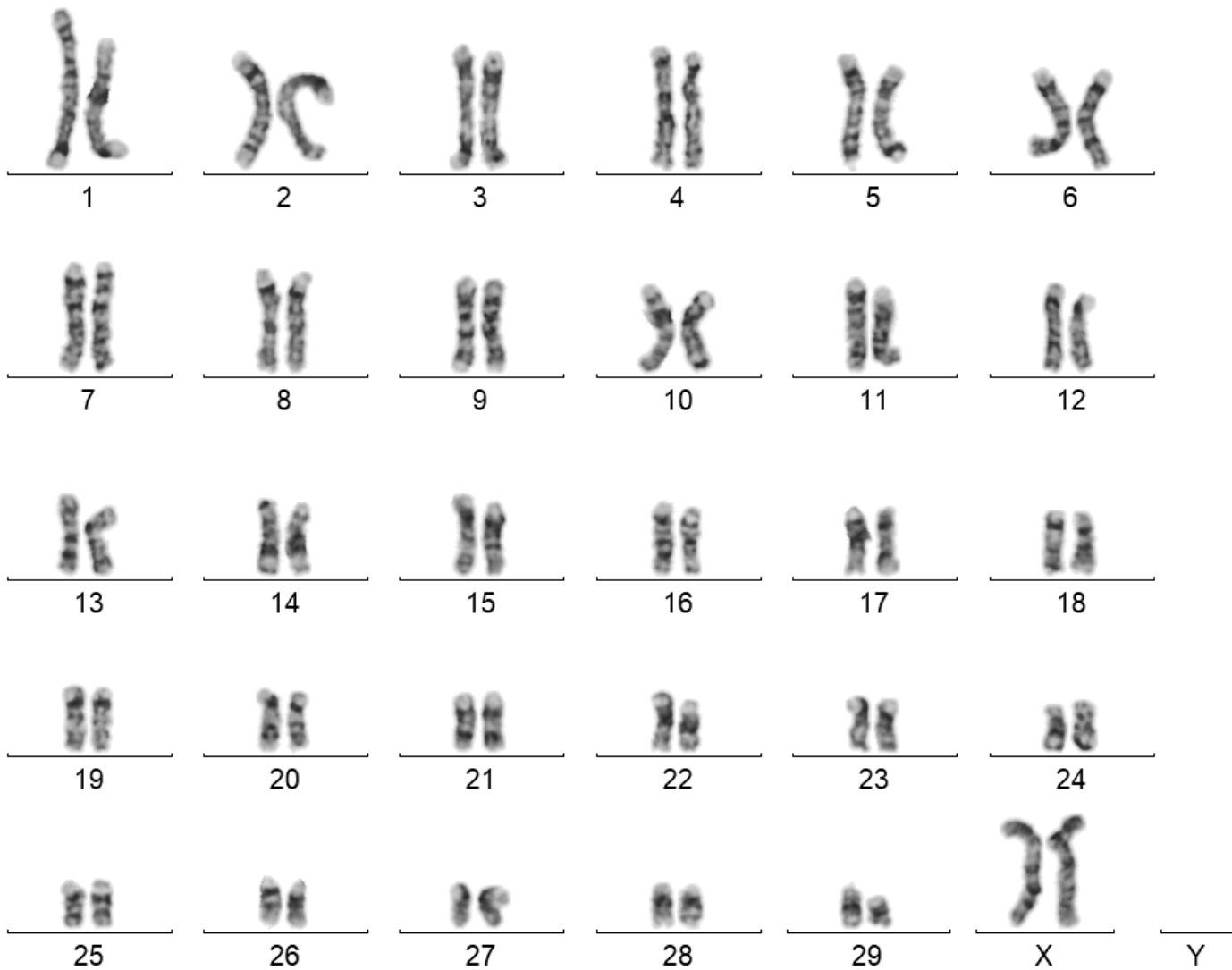

60,XX

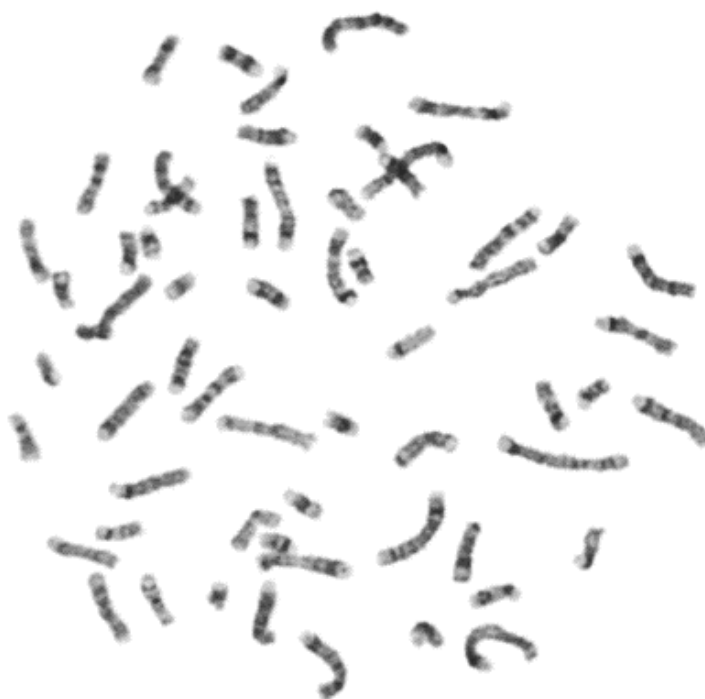

Supplement: jkad156_Supplementary_Data [file jkad156_supplementary_data.zip › Figure_S1_G3-2023-404214.pdf]
